# The Location of the Center of Pressure on the Starting Block Is Related to Sprint Start Performance

**DOI:** 10.3389/fspor.2019.00021

**Published:** 2019-09-06

**Authors:** Ryu Nagahara, Yuji Ohshima

**Affiliations:** ^1^National Institute of Fitness and Sports in Kanoya, Kanoya, Japan; ^2^Institute for General Education, Ritsumeikan University, Kusatsu, Japan

**Keywords:** block clearance, GRF, running, acceleration, power, track and field

## Abstract

Force application locations [i.e., center of pressure (COP)] on the block surface are not necessarily the same for individuals even if the same block locations and angles are used. The purpose of this study was to examine the association of block clearance performance with COP location on the starting block surface. Twenty-one male sprinters performed 60 m sprints from the starting blocks, during which the ground reaction forces (GRFs) on the starting blocks were recorded using two force platforms. Using a previously validated method, changes in COP location on the block surface during the block clearance for each block was calculated from the marker coordinates on the block surface, GRF signals, and moment data around the center of the force platform at the ground level. Moreover, average horizontal external power (AHEP), which was considered the key performance criterion, was computed. Statistical parametric mapping (SPM) 1D linear regressions were used to test relationships between AHEP and COP location curves in the anteroposterior and vertical directions. The COP for both legs moved backward and upward (0.042 and 0.042 m for the front block and 0.030 and 0.034 m for the rear block) at first and then forward and downward (0.113 and 0.094 m for the front block and 0.095 and 0.087 m for the rear block) toward the toe-off. Based on SPM results, AHEP was correlated with front block anteroposterior and vertical COP locations from 12.9 to 20.8% and from 10.4 to 22.2% of the force production phase, respectively, while it was correlated with rear block vertical COP location from 31.9 to 37.4% of the force production phase. In conclusion, the current results demonstrate that, regardless of the starting block location and angle, better sprint start performance is accomplished with a higher and more to the rear COP on the starting block surface, when COP is located close to heel during the middle phase of the block clearance. The fact that the COP location is related to sprint start performance will be useful for sprinters and coaches who intend to improve sprint start performance.

## Introduction

Block clearance at the start of a race is important for the entire performance of a 100 m race (Mero, 1988; Bezodis et al., 2015; Willwacher et al., 2016). The magnitudes of net and propulsive GRFs at the block clearance are pivotal for a better sprint start performance (Rabita et al., 2015; Bezodis et al., 2019). To produce large net and propulsive GRFs during the sprint start, starting blocks are used for the start of sprint races.

The location and angle of each block can be arranged by a sprinter for accomplishing his/her best race performance. Because of these regulations, there have been studies which examined locations and angles of starting blocks for better start or entire sprint race performances (Dickinson, 1934; Kistler, 1934; Henry, 1952; Sigerseth and Grinaker, 1962; Stock, 1962; Guissard et al., 1992; Schot and Knutzen, 1992; Harland and Steele, 1997; Mero et al., 2006; Slawinski et al., 2012; Schrödter et al., 2016). For example, it has been found that longer anteroposterior inter-block spacing could result in greater block clearance velocity through greater propulsive impulse, if it was accompanied with longer push phase duration and vice versa (Dickinson, 1934; Kistler, 1934; Henry, 1952; Schot and Knutzen, 1992). Moreover, intermediate anteroposterior inter-block spacing was recommended for better block clearance performance (Sigerseth and Grinaker, 1962; Stock, 1962). For block angles, no effect of habitual block angle on block power was found (Schrödter et al., 2016), whereas the reduction of the front block angle resulted in an increment of block clearance velocity with consistent block clearance duration (Guissard et al., 1992; Mero et al., 2006).

Although the above-mentioned findings are useful for understanding determinants of the block clearance performance in terms of a block location and angle, actual force application locations [i.e., center of pressure (COP)] on the block surface are not necessarily the same for individuals even if the same block locations and angles are used. Thus, the COP location on the starting block surface during the block clearance would be a new interesting aspect of the performance indicator for block clearance. Moreover, such information will be beneficial for understanding better strategy of force application on the starting blocks. In addition, the location of COP on the starting block surface for better start performance will be useful for sprinters to manipulate the location of force application on the starting block during the block clearance. A method calculating COP location on the starting block surface has recently been proposed, and its accuracy has been confirmed (Ohshima et al., 2019). Typically, COP on the starting block moves backward and upward at first and then forward and downward until toe-off for both the front and rear legs (Ohshima et al., 2019). Using this new method, the actual COP location on the block surface for better performance can be examined. Moreover, changes in COP location on the starting block surface have only been reported for two sprinters, and the general shape of changes in COP location for sprinters is still unknown. Elucidating changes in COP on the starting block surface would provide insight into the force production manner of one of the most powerful human movements.

The purpose of this study was to examine the association of block clearance performance with COP location on the starting block surface. Knowledge gained from the examination of the association of the block clearance performance with COP locations would be useful for sprinters and coaches when they try to improve block clearance performance.

## Materials and Methods

### Participants

Twenty one male sprinters (mean ± SD: age, 20.4 ± 1.4 y; stature, 1.73 ± 0.06 m; body mass, 65.7 ± 4.3 kg; personal best 100-m time, 11.24 ± 0.41 s) participated in this study. Before the experiment, all participants were fully informed of the aim, risks of involvement, and experimental conditions of the study, and gave their written consent. This study was approved by the research ethics committee of the institute.

### Experiments

After warming up, the participants wearing their own spiked shoes performed two maximal effort 60 m sprints from starting blocks with a rest period of 10 min between the trials. The toes of all the participants did not touch the ground and were located at the front edge of the block surface. Two force platforms (TF-32120, Tec Gihan, Uji, Japan; 1,000 Hz), which can measure forces applied by feet separately during block clearance, were used to measure the ground reaction forces (GRFs). A starting block rail (Super III NF155B, Nishi, Tokyo, Japan), which is permitted for use in official races, was bolted at four locations to the force platform covered by athletic track surface (see Ohshima et al., 2019 for detail). Thus, the block itself could be relocated easily, and in exactly the same ways as it could in a race. Sprint time at the 10-m mark was measured using a photo-cell system (TC Timing System, Brower, Draper, UT, USA), and an electric starting gun connected to an operating computer of force platforms provided the start signal, and initiated the timer and recording of GRF.

**Figure 1:**
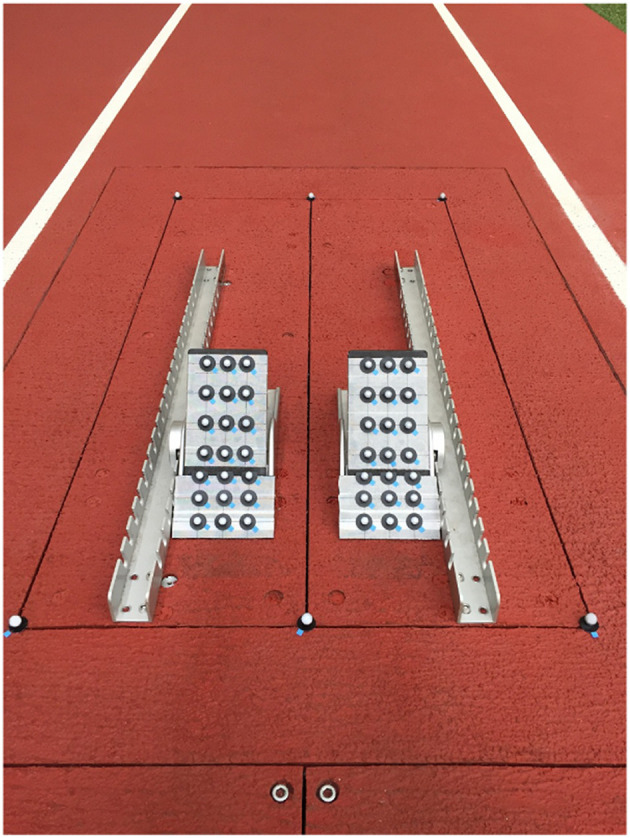
Depiction of the experimental set-up for obtaining coordinates of 21 markers on each of the starting blocks for the COP calculation including the force platforms, starting blocks and rails, and markers on the starting blocks.

Twenty one small retro-reflective markers (11 mm in diameter) were affixed to the surface of each starting block (Figure 1). Before the trials, the locations of the markers on the starting block surface, which are necessary for coordinate transformation, were determined using a motion capture system (Raptor-E, Motion Analysis Corporation, Santa Rosa, CA, USA; 100 Hz, 10 cameras) for all combinations of block locations and angles (17 locations and five angles) for each block (see Ohshima et al., 2019 for detail). The affixed markers on the starting block surface were removed after the coordinates were recorded. Through this procedure, COP in any combination of block locations and angles could be calculated. The location and angle of each block for each participant was recorded at the trial. The block location and angle of the two blocks were arranged by each participant for his suitable locations and angles.

### Data Processing

Based on the 10 m time, the fastest trial for each participant was adopted for the following data processing. GRF signals were smoothed with a fifth-order spline filter (Woltring, 1986). The cut-off frequency was 50 Hz (Nagahara et al., 2017, 2018a,b). Using a previously validated method (Ohshima et al., 2019), changes in COP location on the block surface during the block clearance for each block was calculated by a simple coordinate transformation using the obtained marker coordinates, GRF signals and moment data around the center of the force platform at the ground level. Briefly, COP values were calculated by separating the starting block surface into six tandem parts, using each of three markers on the block surface for the block coordinate system. In the case of the lower part, the origin of starting block coordinate system was set at the lowest and far-right marker on the block surface in Figure 1, while the block coordinate system was made adopting the other two markers which were located at the lowest and far-left and at the second lowest and far-right, respectively, on the starting block. Using a simultaneous equation shown below, COP on the block surface in the global coordinate system was calculated.

(1) $$\begin{array}{l} \left(\left[\begin{array}{c} {\vec{r}}_{x}^{OB}\\ {\vec{r}}_{y}^{OB}\\ {\vec{r}}_{z}^{OB} \end{array}\right]\;+\;\left[\begin{array}{ccc} a_{1,1} & a_{1,2} & a_{1,3}\\ a_{2,1} & a_{2,2} & a_{2,3}\\ a_{3,1} & a_{3,2} & a_{3,3} \end{array}\right]\;\cdot \;\left[\begin{array}{c} {\vec{r}}_{x}^{BP}\\ {\vec{r}}_{y}^{BP}\\ {\vec{r}}_{z}^{BP} \end{array}\right]\right)\;\times \;\left[\begin{array}{c} O_{fx}\\ O_{fy}\\ O_{fz} \end{array}\right]\\ \;+\left[\begin{array}{ccc} a_{1,1} & a_{1,2} & a_{1,3}\\ a_{2,1} & a_{2,2} & a_{2,3}\\ a_{3,1} & a_{3,2} & a_{3,3} \end{array}\right]\;\cdot \;\left[\begin{array}{c} 0\\ 0\\ B_{n_{z}^{couple}} \end{array}\right]=\left[\begin{array}{c} O_{n_{x}^{total}}\\ O_{n_{y}^{total}}\\ O_{n_{z}^{total}} \end{array}\right] \end{array}$$

where ${\vec{r}}_{x}^{OB}$, ${\vec{r}}_{y}^{OB}$, and ${\vec{r}}_{z}^{OB}$ are coordinates of the origin of the starting block coordinate system (*B*) in the force platform (global) coordinate system (*O*), in which the origin is set at the center of force platform at ground level; *a*_1, 1_ to *a*_3, 3_ are the components of a coordinate transformation matrix of the starting block coordinate system (*B*) to the force platform coordinate system (*O*); ${\vec{r}}_{x}^{BP}$, ${\vec{r}}_{y}^{BP}$, and ${\vec{r}}_{z}^{BP}$ are the coordinates of the COP (*P*) in the starting block coordinate system (*B*); ${\;}^{O}f_{x}$, ${\;}^{O}f_{y}$, and ${\;}^{O}f_{z}$ are applied forces onto the ground in the force platform coordinate system (*O*); ${\;}^{B}n_{z}^{couple}$ is the free moment applied on the x'y' (block surface) plane of the starting block coordinate system (*B*); and ${\;}^{O}n_{x}^{total}$, ${\;}^{O}n_{y}^{total}$, and ${\;}^{O}n_{z}^{total}$ are applied moments around the origin of the force platform coordinate system (*O*). In the case where the COP (*P*) is on the x'y' plane of the starting block coordinate system (*B*), ${\vec{r}}_{z}^{BP}$ is equal to zero. When the COP moved below the origin of the used coordinate system, the coordinate system for calculating the COP was changed to the lower one.

The onset of the force production and toe-off for each leg were determined using the first derivative of the GRF applied perpendicularly to the block surface with a threshold of >500 N/s (Brazil et al., 2017). Toe-off was defined when the GRF applied perpendicularly to the block surface next fell below 50 N (Brazil et al., 2017). Horizontal velocity was calculated integrating mass-specific filtered anteroposterior GRF with adjusting the influence of air resistance in accordance with previous studies (Colyer et al., 2018; Nagahara et al., 2019). Horizontal velocity was combined with support duration to provide average horizontal external power (AHEP), which was considered the key performance criterion, in reference to Bezodis et al. (2010). AHEP was divided by body mass.

### Statistical Analyses

Descriptive data were presented by means and standard deviations (SDs). The correlation coefficient was calculated to examine relationships between AHEP and COP discrete variables. Statistical parametric mapping (SPM) 1D linear regressions were used to test relationships between AHEP and COP location curves in the anteroposterior and vertical directions (Pataky, 2012). The significance level was set at *p* < 0.05. Threshold values for the interpretation of correlation coefficient as an effect size were 0.1 (small), 0.3 (moderate), 0.5 (large), 0.7 (very large), and 0.9 (extremely large) (Hopkins et al., 2009).

## Results

**Figure 2:**
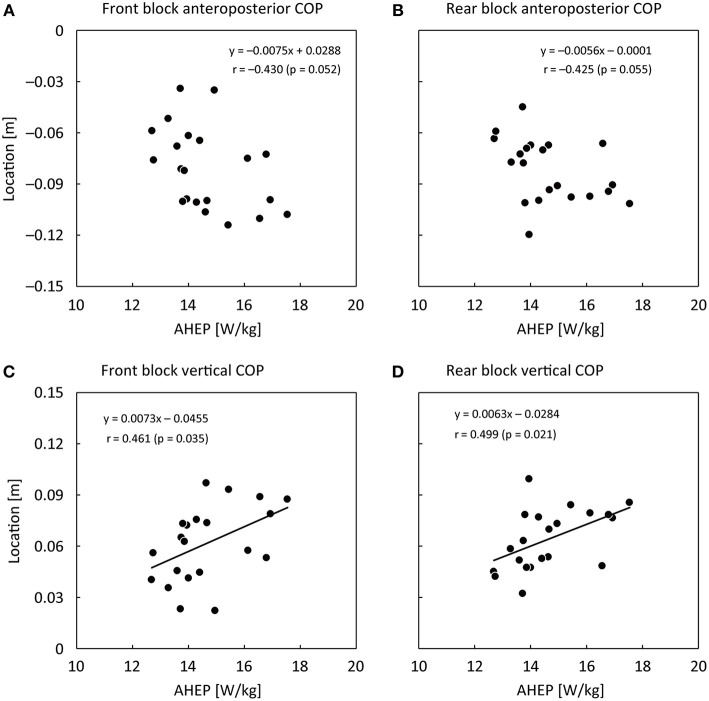
Relationship of AHEP at the start with the front block mean anteroposterior COP location **(A)**, the rear block mean anteroposterior COP location **(B)**, the front block mean vertical COP location **(C)**, and the rear block mean vertical COP location **(D)**.

**Figure 3:**
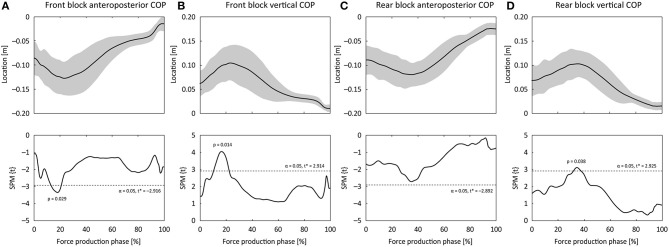
Normalized mean COP curves during the force production phase at the block clearance, and the associated SPM-1D *t*-test results for association of average horizontal external power with each COP location. **(A)** Front block anteroposterior COP, **(B)** Front block vertical COP, **(C)** Rear block anteroposterior COP, **(D)** Rear block vertical COP. The second row of the panels shows SPM-1D linear regression test results. Ranges where the curve being above or below the dotted line indicate statistically significant differences between curves.

The 10 m time was 2.09 ± 0.07 s. COP locations (the origin is at the middle of two blocks, the front edge of each block and the ground level) and starting block locations (anteroposterior distance between the starting line to the front edge of the block surface), and angles (from ground level) were shown in Table 1. For the COP locations on the block surface, mean COP locations in the vertical direction for both legs were positively correlated with AHEP (moderate effect), whereas there were no correlations between the mean COPs in the mediolateral and anteroposterior directions and AHEP (Table 1 and Figure 2). Figure 3 shows changes in COP locations in the anteroposterior and vertical directions for the front and rear blocks and the results of the SPM analyses. The COP for both legs moved backward and upward (0.042 and 0.042 m for the front block and 0.030 and 0.034 m for the rear block) at first and then forward and downward (0.113 and 0.094 m for the front block and 0.095 and 0.087 m for the rear block) toward the toe-off. The timing of moving COP shifting to forward and downward was earlier in the front block (Figure 3). Based on SPM results, AHEP was correlated with front block anteroposterior and vertical COP locations from 12.9 to 20.8% and from 10.4 to 22.2% of the force production phase, respectively, while it was correlated with rear block vertical COP location from 31.9 to 37.4% of the force production phase (Figure 3).

**Table 1:** Mean and SD for AHEP, COP locations and block locations and angles, and relationship of AHEP with other variables.

**Variables [units]**	**Mean ± SD**	**Correlation coefficient (*P*-value)**
AHEP [W/kg]	14.7 ± 1.4	
Front block mediolateral mean COP location [m]	0.098 ± 0.007	0.237 (0.301)
Front block anteroposterior mean COP location [m]	−0.080 ± 0.024	−0.428 (0.052)
Front block vertical mean COP location [m]	0.061 ± 0.022	**0.461 (0.035)**
Rear block mediolateral mean COP location [m]	0.098 ± 0.007	−0.094 (0.686)
Rear block anteroposterior mean COP location [m]	−0.082 ± 0.018	−0.423 (0.055)
Rear block vertical mean COP location [m]	0.064 ± 0.018	**0.499 (0.021)**
Front block location [m]	−0.45 ± 0.05	0.077 (0.739)
Front block angle [°]	48.9 ± 3.8	0.099 (0.669)
Rear block location [m]	−0.69 ± 0.06	−0.144 (0.533)
Rear block angle [°]	53.0 ± 3.5	−0.070 (0.763)

The mediolateral COP values for both sides are shown in positive values.

AHEP, average horizontal external power; COP, center of pressure.

*Bold values indicate significant correlations*.

## Discussion

This study is the first to investigate whether COP locations on the starting block surface are related to sprint start performance. The main findings were that (1) there were positive correlations between the mean vertical COP locations on the starting block surface and AHEP, and (2) the COP locations were correlated with AHEP where the COPs were located at the high and rear on the starting block surface during the force production phase.

The higher mean vertical location of COP on the starting block surface was correlated with greater AHEP for both legs. Moreover, although correlation coefficients between AHEP and the mean anteroposterior location of COP on the starting block surface for both legs came short of the significance level, effect sizes of the corresponding relationships were moderate and were the same as the effect size of the relationships between AHEP and the mean vertical locations of COP on the starting block surface. In contrast, the location and angle of the blocks did not show correlations with AHEP. These results demonstrate that higher and possibly more to the rear COP location on the starting block is advantageous for better sprint start performance regardless of starting block locations and angles. Moreover, COP locations were only correlated with AHEP where the COPs were located at the high and rear on the block surface, indicating that COP location can be a determinant of sprint start performance when it is relatively close to heel, while COP at the initial or terminal location is not decisive for better sprint start performance. Accordingly, COP location on the starting block should be taken into account for achieving better sprint start performance. In order to accomplish the higher and more to the rear COP on the block surface, when COP is located close to heel during the middle phase of the block clearance, suppressing force production at the fore-foot through suppressing ankle plantar flexion during the initial force production duration will possibly be a useful way for sprinters.

The current finding of no correlation between AHEP and block angles are in line with a previous study which revealed no effects of habitual block angle on block power (Schrödter et al., 2016). For the COP locations on the starting block surface, it moved backward and upward at first and then forward and downward toward the toe-off for both legs, and these changes in COP location on the starting block surface are consistent with a previous case report (Ohshima et al., 2019). Moreover, this backward movement of COP on the starting block surface before forward movement is similar to the movement of COP during the vertical jump (Le Pellec and Maton, 2002). Because no study has investigated the relationship of COP location on the starting block surface with the start performance, it is difficult to compare the current results to previous studies.

Higher COP location on the starting block surface will make it possible to shorten the vertical distance between the GRF vector and the whole body center of mass which allows sprinters to efficiently produce the propulsive force, resulting in a better block clearance performance. From the other aspect, higher and more to the rear COP location on the starting block surfaces means that the COP is closer to the ankle joint, indicating that the distance between force application location and the ankle joint center is short. This shorter distance between application location and the ankle joint center allows a sprinter to produce the force on the block with smaller ankle plantar flexion moment, which may possibly enhance the efficiency of force transmission from hip and knee to the ground. The magnitudes of net and propulsive GRFs at the block clearance are decisive for better sprint start performance (Rabita et al., 2015; Bezodis et al., 2019). Moreover, based on the force-time curves, time spans where the correlations were found using SPM analyses are approximately the ranges where the forces rapidly develop. Taken together, the higher and more to the rear COP on the starting block surface, when COP is located close to heel during the middle phase of the block clearance, is likely efficient for producing the large magnitude of force onto the starting block, and this might result in the correlation of AHEP with COP locations.

There are some limitations on the findings of this study. Because a foot location was not recorded in this study, the relationship between foot and COP locations is still unknown. Moreover, relationships of body segment configurations with COP locations and sprint start performance will be an interesting topic of a future study. The participants in this study were only male sprinters and not international level, and thus it is possible that a different conclusion would be derived from hurdlers, female or elite sprinters.

In conclusion, the current results demonstrate that, regardless of the starting block location and angle, better sprint start performance is achieved with a higher and more to the rear COP on the starting block surface when COP is located close to heel during the middle phase of the block clearance. The fact that the COP location is related to sprint start performance will be useful for sprinters and coaches who intend to improve sprint start performance.

## Data Availability

The datasets generated for this study are available on request to the corresponding author.

## Ethics Statement

The studies involving human participants were reviewed and approved by Research ethics committee of the National Institute of Fitness and Sports in Kanoya. The participants provided their written informed consent to participate in this study.

## Author Contributions

RN and YO contributed to conceiving, designing, and performing the experiment, to analyzing the data, and to drafting and revising the article. RN performed most of the data analysis and drafting the article.

### Conflict of Interest Statement

The authors declare that the research was conducted in the absence of any commercial or financial relationships that could be construed as a potential conflict of interest.
